# *In Silico *cancer cell versus stroma cellularity index computed from species-specific human and mouse transcriptome of xenograft models: towards accurate stroma targeting therapy assessment

**DOI:** 10.1186/1755-8794-7-S1-S2

**Published:** 2014-05-08

**Authors:** Xinan Yang, Yong Huang, Younghee Lee, Vincent Gardeux, Ikbel Achour, Kelly Regan, Ellen Rebman, Haiquan Li, Yves A Lussier

**Affiliations:** 1 Ctr for Biom. Info. Section of Genetic Medicine, Dept. of Medicine, U. of Chicago, IL, USA; 2 Institute for Translational Health Informatics, U. of Illinois at Chicago, IL, USA; 3 Department of Informatics, EISTI engineering school, Cergy, France; 4 Dept. of Medicine, U. of Illinois at Chicago, IL, USA; 5 Comprehensive Cancer Ctr and Ludwig Ctr for Metastasis Research, U. of Chicago, IL, USA; 6 Section of Hematology/Oncology, Department of Pediatrics, U. of Chicago Medicine Comer Children's Hospital, IL, USA; 7 Depts of Bioengineering & of Pharmaceutical Science, U. of Illinois at Chicago, IL, USA; 8 Comp. Inst. & Inst. for Genomics & Syst. Bio., Argonne Nat. Lab. and U. of Chicago, IL, USA; 9 Inst. for Personalized Respiratory Medicine, U. of Illinois at Chicago, IL, USA; 10 This work was conducted in part while at The University of Chicago, IL, USA; 11 Cancer Ctr, BIO5 Inst., Clinical & Translational Science Inst., U. of Arizona, AZ, USA

## Abstract

**Background:**

The current state of the art for measuring stromal response to targeted therapy requires burdensome and rate limiting quantitative histology. Transcriptome measures are increasingly affordable and provide an opportunity for developing a stromal versus cancer ratio in xenograft models. In these models, human cancer cells are transplanted into mouse host tissues (stroma) and together coevolve into a tumour microenvironment. However, profiling the mouse or human component separately remains problematic. Indeed, laser capture microdissection is labour intensive. Moreover, gene expression using commercial microarrays introduces significant and underreported cross-species hybridization errors that are commonly overlooked by biologists.

**Method:**

We developed a customized dual-species array, **H&M array**, and performed cross-species and species-specific hybridization measurements. We validated a new methodology for establishing the stroma vs cancer ratio using transcriptomic data.

**Results:**

In the biological validation of the H&M array, cross-species hybridization of human and mouse probes was significantly reduced (4.5 and 9.4 fold reduction, respectively; p < 2x10^-16^ for both, Mann-Whitney test). We confirmed the capability of the H&M array to determine the stromal to cancer cells ratio based on the estimation of cellularity index of mouse/human mRNA content *in vitro*. This new metrics enable to investigate more efficiently the stroma-cancer cell interactions (e.g. cellularity) bypassing labour intensive requirement and biases of laser capture microdissection.

**Conclusion:**

These results provide the initial evidence of improved and cost-efficient analytics for the investigation of cancer cell microenvironment, using species-specificity arrays specifically designed for xenografts models.

## Background

In spite of developments in high throughput molecular assessment biotechnologies, it remains particularly challenging to investigate molecular expression in the tumor microenvironment containing local tumor stoma, reactive stromal cells and cancer cells. There is increasing evidence that the development and progression of cancer is significantly affected by interactions between the tumor and its microenvironment [1-6], suggesting that the ability to profile gene expression in both stromal and cancer cell compartments is critical. Malignant tumors are composed of cancer cells and their associated host cells, which include blood and lymph endothelial cells, immune cells, fibroblasts and myofibroblasts [7,8]. The host cells, also termed stromal cells here, make up about half of most malignant tumors [9] and have emerged as targets of anti-tumor therapy in recent years [10].

Currently, the best way to selectively isolate cancer cells from a heterogeneous population of cells in a tumor is Laser Capture Microdissection (**LCM**) [11]. While LCM is increasingly used in the selection of cancer cells [12-14], the application of LCM to stromal masses presents several important technical challenges: (i) some cell types (e.g. endothelial cells) are too long and thin or intertwined with cancer cells to be isolated due to their infiltrating growth and (ii) the visualization of samples and accurate determination of cell type can be difficult because cellular staining via immunochemistry is not compatible with array analyses [10]. Furthermore, LCM of large numbers of cells from many sections and samples is rate limiting and cumbersome, requiring a considerable amount of time (1 day/sample) and sub-zero temperatures. Therefore, novel and affordable high throughput approaches are required to assess the expression of cancer and stromal cells to determine their molecular interactions. Further, transcriptome measures from RNA-seq or conventional expression arrays present the opportunity to develop *in silico *estimates of cancer cells versus stromal cell ratios, which otherwise require labour intensive quantitative histology measures across hundreds of microscopic fields per tissue sample.

Xenograft models, where human cancer cells are grown in immunodeficient mice, are popular for studying the tumor microenvironment. In such models, the genes of the stromal compartment and cancer cells come from distinct species: mouse and human respectively. While gene expression profiles of human cancer and mouse stroma have been analysed by human microarrays [15], this approach is limited because the human and mouse genomes are highly homologous and commercially available arrays are not designed for simultaneously measuring both mouse and human mRNAs [16]. Thus, the interpretation of transcriptome intensities is confounded by **Cross-Species Hybridization (CSH)**, which we have biologically confirmed to exist between human array probes with universal mouse RNAs [17]. We also predicted that this CSH in xenograft models would preferentially occur among homologous human-mouse genes in human arrays resulting in combined gene expression signals where deregulated mouse stromal genes are jointly measured along with human cancer genes [17]. We previously hypothesized that deregulated probes on human arrays exposed to whole xenograft tissue experiments could be mined to identify those probes most likely harbouring human-mouse CSH (due to homologous genes and alternative non-homology factors), and that we could impute the enrichment of biological processes and molecular functions of stromal mechanisms via these CSH probes using Gene Ontology (GO) gene set annotations [17]. While many probes of homologous genes can be enriched for a GO signal, a limitation of this previous study is that this *geneset*-based approach is not designed to impute differential expression of *individual *mouse stromal genes. There is thus a need for improved species specificity of probes in newer arrays designed specifically for jointly studying stromal and cancer cell gene expression in xenograft models.

In many tumor biology and pharmacology xenograft studies, the interplay and co-expression patterns of cancer and stromal cell genes need to be studied using the simultaneous analysis of both species in the same array experiment. When tissue expression is detected using separate mouse and human arrays, comparing mouse and human gene expression requires complex and artefact-inducing normalization of the two datasets. Currently, there is no genome-wide dual-species microarray available. While whole transcriptome RNA-sequencing (RNA-seq) of xenograft models provides an elegant alternative, the problem of disambiguating the expression intensities of homologuous genes remains (though the solution is quite different: an ambiguous RNA assembly problem). There exist two published dual-species partial arrays, in which one contains a mere 516 human probe-sets and 456 mouse probe-sets [16] and a second contains only protease and inhibitor genes [18]. Previously, we reengineered the process of microbial diagnosis by designing a Panmicrobial array that contained 9,477 species specific probes (SSPs) to address 1,710 distinct vertebrate viruses [19-21]. Drawing from this experience, we hypothesized that we could establish a comprehensive map of CSH in human and mouse arrays. We thus designed the first genome-wide human-mouse dual-species array (H&M array) for comprehensively investigating gene expression of stromal. Furthermore, we also hypothesised that this new dual species array would allow for the development of a novel estimation of the stroma/cancer cell ratio via a cellularity index based on SSPs of homologous housekeeping genes as an alternative to traditional histological LCM assessments. Here we report the first genome-wide dual species array for human and mouse gene expression in xenograft models and identify novel biological functions for CSH probes, that until now have been misattributed to either human cancer cells or mouse stromal cells.

## Methods

### Datasets and custom human-mouse whole genome arrays used in this study

Microarray data from the two *in vitro *experiments (Table 1) are available in the GEO repository under accession number GSE23377 (initial *in vitro *biological experiment: GSE23054, version 1 GEO platform: GPL10714; *in vitro *validation experiment for CSH and stroma cell ratio estimation: GSE23364, version 2 GEO platform: GPL10749, **Agilent Custom Array Order #023265**). We used nine genomic datasets to compute human-mouse CSH (Additional file 1**- Supp. Table S1)**. The list of housekeeping mouse genes was previously published [22,23] and the list of homologous mouse-human genes was downloaded from NCBI (ftp://ftp.ncbi.nih.gov/pub/HomoloGene) on Feb, 2009 (Build 63). By design, these experiments generated **Datasets S1, S2 and S3 **that we provide for reuse by other groups at http://lussierlab.org/publications/HsMm_array

**Table 1:** Cross-species hybridization experiments for H&M array

Initial experiment**						
Dual channel array	1			2		
Exposed to RNA in red channel	1.5ug Hs* RNA (sample 1)			1.5ug Mm* RNA (sample 2)		
Exposed to RNA in green channel	0.75ug Hs* RNA + 0.75 Mm*			0.75ug Hs* RNA + 0.75 Mm*		
Array design	Agilent 2x105k arrays					
**Validation experiment**						
Single channel array	1	2	3	4	5	6
Exposed to RNA	human	human	human	mouse	mouse	mouse
Array design	46k Human probes and 46k mouse probes					

*Hs: Homo Sapiens, Mm: Mus Musculus

**In the initial experiment, two human-mouse dual-species arrays were custom printed on Agilent 2x105k arrays. Each array contained only one species of universal mRNAs on the red channel (either human or mouse) and had a control sample containing equal quantities of universal human and mouse mRNAs on the green channel.

### H&M array design, annotation, and analysis (Figure 1: panels A-C)

**Figure 1:**
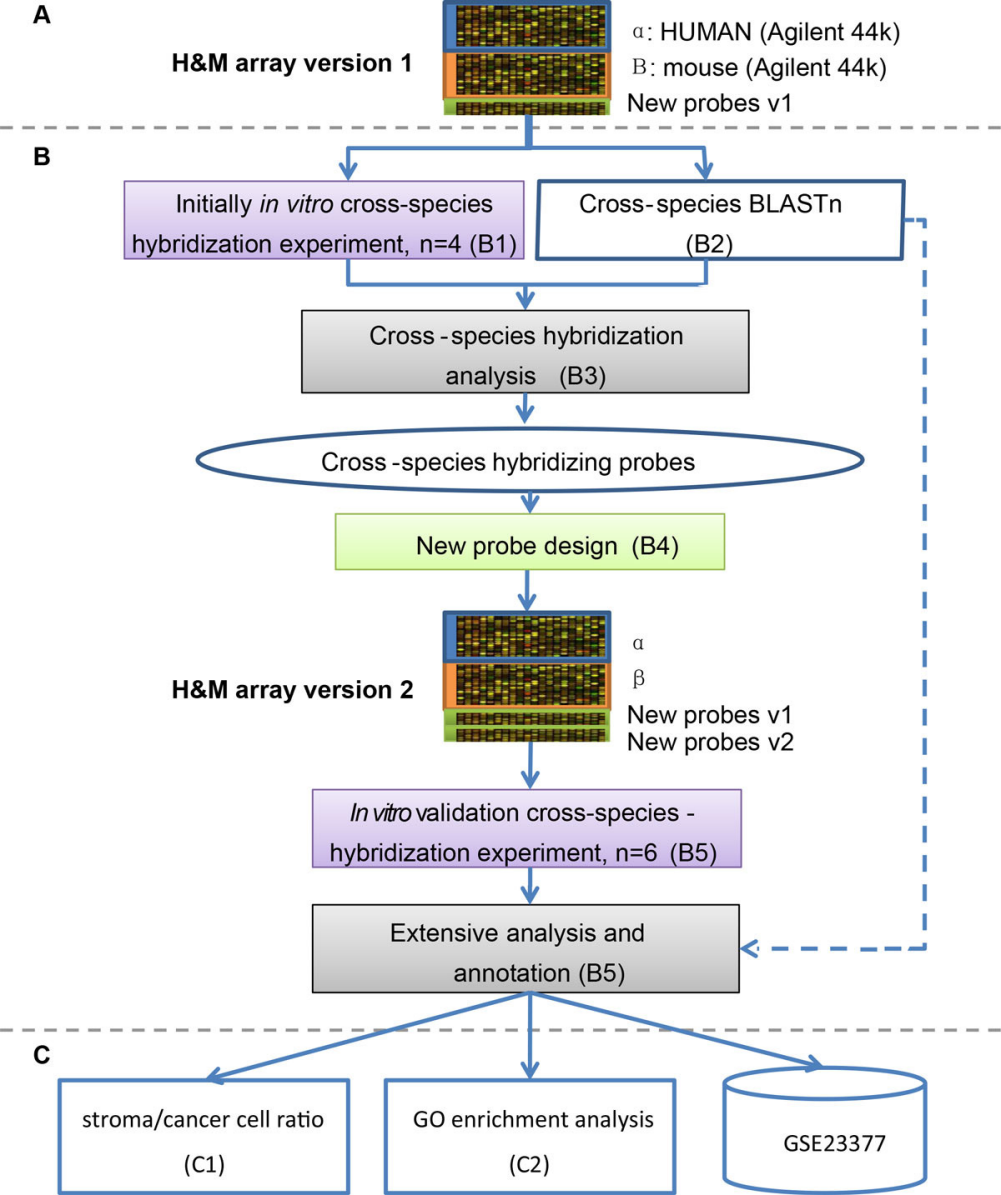
**Schematic diagram for the biological and computational design and annotation of the H&M microarray**. The whole array design and analysis includes three stages, as described in the Materials and Methods Section. There were 3.6k newly designed probes in step A and 7.1k newly designed probes in step B. All probes on H&M (Agilent GEO platform GPL10749) were optimally and extensively annotated for cross-species hybridization as shown in **Dataset S1**. The Agilent pre-annotated cross-species hybridization probes from step A are given in **Datasets S2-3**, and the processes used to design the new probes in version 1 were previously published [17].

As shown in Figure 1, the experimental design consists first of an assemblage of the two sets of 44k probes based on Agilent's commercial human array and mouse array. Agilent also provided cross hybridizing estimates between mouse probes and human mRNAs and vice versa (7141 human and 6420 mouse probes; **Datasets S2, S3**), which are redesigned to correct for comprehensive CSH. We designed two custom arrays: the second and final one improved on the original design after biological validation. The H&M arrays contain all legacy probes from the commercial human and mouse arrays as well as the newly designed ones (Figure 1, **panel A**). The second and final custom H&M array is identified as "H&M array version 2" (Agilent GEO platform: GPL10749; **Agilent Custom Array Order #023265**). In an initial *in vitro *biological experiment, we cross-hybridize H&M array version 1 with mouse mRNAs, human mRNAs, and a combination of both human and mouse RNAs (Figure 1, Step B1; Table 1). We also conduct a parallel and extensive BLASTn analysis of potential cross species hybridization of probes (Step B2). Taken together the biological and the computationally derived cross hybridizations serve as a model for CSH propensity of probes, and have an extensive optimization method (Step B3). As shown in panel B of Figure 1, we conduct a second iteration of probe design (Step B4), and produce version 2 of the H&M array. A second and more comprehensive biological experiment that cross hybridizes six arrays (H&M array version 2) with mouse and human mRNAs is used for the final evaluation (Step B5; Table 1) and improved the annotation of the CSH of each probe (**Datasets S2, S3**). Gene Ontology enrichment studies are also performed and show rectification of the cross-hybridization and validation of cellularity ratios (Figure 1, **panel C**).

### Step B1: initial cross-hybridization experiment of H&M array version 1

As shown in Table 1, each channel of the two H&M arrays is cross-hybridized producing four readouts. Biological experiments are conducted to produce physical evidence of human-mouse cross-hybridization and are designed to identify CSH by independently arraying mouse mRNAs and human mRNAs (in the red channel) against the joint human and mouse probes of the dual-species array. Using dual channel technology (measuring two groups of mRNA labelled with different colors simultaneously on the same array), we also pool human and mouse mRNA in equal parts as a control on the green channel.

We first obtain human and mouse universal reference RNAs from ArrayIt (Sunnyvale, CA), which were derived from a homogenate of all tissues from the organism, making them somewhat representative of any cell type. This universal RNA is amplified and labelled according to the Agilent Low RNA input Fluorescent Linear Amplification Kit protocol and according to Agilent's two-color quick amp labelling protocol. The two arrays are scanned with 5 µm resolution and 100% power gain at 600PMT for both Cy3 & Cy5 using the GenePix 4000B Axon Scanner.

We quantify gene expression values using Agilent Feature Extraction Software and subtract the adjusted background for each probe from the mean expression value. Finally, we use an inclusion criterion to identify the probes that show some RNA binding where the minimum expression intensity has to be larger than a value of "1" when exposed to the pooled human and mouse RNA in both arrays. This threshold was set because when the expressed intensity was less than 1, probes vary widely across the two experimental repeats using the same quantity of pooled human and mouse RNAs.

### Step B2: BLASTn analysis of cross species hybridization in array probes

The Basic Local Alignment Search Tool (BLAST) algorithm [24] is run with default parameters to theoretically predict the possibility of CSH for each probe by comparing human array probes with mouse RNAs, and mouse array probes with human RNAs. BLASTn was used because of its ability to find "fuzzy" alignments to related nucleotide sequences from other organisms. The hits are ranked by their BLASTn score of which the highest scoring hit is recorded, together with (i) the *length of the alignments *and (ii) the number of *mismatches *(Details are given in Table 2 of this manuscript and Figure D in the Suppl. Methods of our previous publication [17]). Further evaluation of the set of parameters used in the optimal prediction model was also conducted (Table 3, Additional file 1**- Supp. Figure S1**).

**Table 2:** Previously established definitions of four biological gold standards (GS) and the parameters of BLASTn results used in the theoretical predictions for identification of CSH probes (mouse probes used as an example).

Gold Standard	Parameters
GS1.*x*	Top *x *ratio of relative expression (red/green) when exposed to erroneous RNA vs to correct RNA:
GS2.x*	Top *x*% absolute expression when exposed to human RNA with mouse probes
GS3.*x*	Top *x*% relative expression when exposed to human RNA with mouse probes (the expression of mouse probes when exposed to human RNA divided by the expression when exposed to mouse RNA)
GS4.*x*	The same as GS2 but absolute log2 based expression on both green channels of human and mouse RNA expression of mouse probes > 4
**Models**	**Conditions and parameters**
Model1*	{18<Align≤20 and mis<5} U {20<Align≤ 30 and mis<6} U {30<Align≤40 and mis<7} U {40<Align≤50 and mis<8} U {50<Align and mis<9}
Model2	{15 < Align ≤ 30 and mis < 2} U {30 < Align ≤ 40 and mis < 3} U {40 < Align ≤ 50 and mis < 5} U {50< Align and mis < 7}
Model3	Alig > 50 & mismatches < 6
Model4	score > 70 & Alig > 58 & mis < 6

**Legend**: biological GS: biological gold standard; CSH: cross-species hybridization; Align: alignment; mis: mismatch; U: union.

The Gold Standards used in the final design are annotated with an asterisks (*). These were selected by comparing the initial biological experimental data and BLAST predictions using H&M array version 1.0 and details of this section are provided in our previous publication [17].

**Table 3:** Definition of two sets of parameters used in the optimal CSH theoretical predictions for identification of CSH probes.

	Conditions	Predicted # of CSH probes for vers.1 mouse probes	Predicted # of CSH probes for vers.1 human probes
Model **1-1**	{18<Alignment<=20 and mismatches<6} UNION {20<Alignment<=30 and mismatches<7} UNION {30<Alignment<=40 and mismatches<8} UNION {40<Alignment<=45 and mismatches<9} UNION {45<Alignment<=50 and mismatches<10} UNION {50<Alignment and mismatches<11}	3819	4755
Model **1-2**	{30<Alignment<=40 and mismatches<8} UNION {40<Alignment<=45 and mismatches<9} UNION {45<Alignment<=50 and mismatches<10} UNION {50<Alignment and mismatches<11}	3525	4370

Computational optimization results shown in Additional file 1 - Supp. Figure S1.

### Step B3: modelling cross species hybridization with biological experiments and sequence alignments

To predict human-mouse cross-hybridizations and further redesign the probes, we optimize both the statistical models for biological measurement (biological Gold Standard - **GS**) and the parameters used in the CSH theoretical prediction models which are compared with the candidate biological GSs in an iteratively optimal way [17]. Each comparison results in an F-score **(Equation 1 **described below after the **Gold Standard) **that balances the recall (sensitivity) and precision and the CSH theoretical prediction model with the largest F-score is selected. Here we present, as an example, our procedure for identifying mouse probes that are most likely to hybridize with human RNAs, the converse would follow similar steps.

• ***CSH theoretical prediction models***: Based on our previously selected theoretical prediction models of cross-hybridization [17] (Table 2, Model 1), we further construct the BLAST prediction model based on the total length of the sequence alignment and the admissible maximum mismatches within the sequence, as shown in Table 3. In order to balance recall and precision while identifying cross-hybridizing probes for which the target gene requires a substitute SSP on the H&M Array, the optimal set of BLAST predictions (*length of the alignments *and the number of *mismatches*), are determined using the ideal F-Score while systematically varying each parameter independently **(**Additional file 1**- Supp. Figure S2)**.

• ***Gold standards derived from the biological experiments***: We stratify the mouse probes exposed to human RNAs according to their level of expression in the biological array experiment (Table 1). For H&M array version 1, using a dual channel experimental design, the background adjusted absolute CSH expression of probes (i.e. the raw, unmodified expression of human probes when exposed to mouse RNAs or the expression of mouse probes when exposed to human RNAs), was stratified and alternate GSs are produced accordingly. The optimal biological GS is determined using relative high F-scores[17]. Since the single channel repeats of H&M Array version 2 allow for the use of a relative ratio of average expression of cross species hybridization over species-specific expression after Variance Stabilization Normalization (VSN) normalization[25], the optimal biological GS was determined using both the relatively higher F-scores and the absolute highest recall.

• ***Accuracy calculation***: True positive CSH probes were those that cross-species hybridized in the biological GS and were also predicted by BLAST to do so. False positives were those that were predicted by BLAST but were not found in the GS. The precisions, recalls (sensitivities) and F-scores (*F*) were calculated for each theoretical model of cross-hybridization against each biological GS at different thresholds of biologically cross-hybridizing probes **(Equation 1)**.

(1) $$F=2\times \frac{precision\times recall}{precision+recall}$$

• ***Identification of cross-hybridizing probes (***Table 3***)***: Once the optimal set of BLAST prediction parameters and biological GSs were determined, all probes from the GS that were considered as CSH were tagged and new SSPs were designed - regardless of whether they were predicted as cross-hybridizing by BLAST. Additionally, probes were tagged for future design when they did not have any expression on the array and were predicted as cross-hybridising using BLASTn program (Table 3: Model 1-1).

### Step B4: probe redesign (green boxes in Figure 1)

**I**. Gene targets associated with cross species hybridizing probes were identified for redesign. Transcripts (RefSeq IDs) associated with each probe were identified from the Agilent microarray annotation file (Additional file 1**- Supp. Table S1**, resources 1-2). For those probes with a retired Refseq or Unigene ID, the batch BLASTn program [24] was used to retrieve the associated antisense Refseq or Unigene sequences. The transcripts targeted by these probes were downloaded from the NCBI GEO database via accession numbers GPL6840 and GPL7202.

**II**. New probes for the identified gene targets were designed using the "GE Probe Design" program in the Agilent eArray (Agilent, Santa Clara, CA) software. The eArray parameters, that we used, include probe length (60nt), probes per target (1), probe orientation (sense) and design options ("best probe methodology" and "design with 3' bias"). These probes were further verified computationally for cross-hybridization.

**III**. Newly designed probes were evaluated by the eArray "Probe Check" program using the built-in transcriptome. The new candidate probes were retained if they did not hybridize within their species with other targets than the intended one nor across species with any other transcripts. Otherwise, these candidate probes were tagged as inappropriate and new probes were designed for the intended target iteratively until they met these criteria.

**IV**. All probes of the commercial arrays were retained, while new probes were also added to the H&M array. Annotation tables of cross species hybridization were used as filters to identify the most SSP for each gene, while the legacy probes were intended to allow for comparison of results with studies using the commercial platforms.

### Step B5: cross-hybridization experiment and extensive annotation of the H&M array version 2

To validate the newly designed probes and extensively annotate the H&M array version 2, a second biological experiment was conducted by hybridizing all the probes on the H&M array version 2 to three human melanoma cell lines (MDA-MB-435) and three universal mouse mRNAs. Six Samples (three with human RNA and three with mouse RNA) were hybridized to the H&M array version 2.0 (Table 1). The subsequent array scanning and image processing followed the same procedure as the initial biological experiment. Additionally, the background-subtracted expression measurements of six arrays were normalized using VSN method [25] and were log2 transformed. Human and mouse probe sets were separately normalized to control for variation in the proportion of human and mouse RNA across xenograft replicates [16]. For each probe, the mean signal intensity of the three biological replicates exposed to the RNAs of the same species was used as the expression value for that species. GS and BLAST parameter optimizations were performed as described in Step 4 and in our previous publication [17], and the propensity for CSH of each probe was annotated (**Dataset S1**). This extensive annotation of CSH was conducted by systematically extending the threshold of the biological GS in 10% increments from 0% to 100% for all probes.

### Stromal cell to cancer cell mRNA ratio estimation (Figure 1, step C-1)

Housekeeping genes are typically constitutively expressed genes required for the maintenance of basal cellular functions, and are thus assumed to be stably expressed under the same experimental conditions as well as between human tissues and mouse tissues [22,23]. To estimate the RNA expression ratio between human and mouse using RNA quantity controlled H&M array experiments, the expression value of human SSPs targeting human housekeeping genes was divided by the expression value of mouse SSPs targeting homologous housekeeping genes.

The SSPs satisfied the following four criteria: 1) they were positively expressed (expressed intensity larger than 1, see Method: Step B1) across all samples when exposed to the intended RNA, 2) among all probes for one species, they expressed below a certain percentage of all expression values when exposed to the RNA of the other species, 3) they were not identified as CSH probes, and 4) were not theoretically predicted as CSH probes by the BLASTn algorithm. **For simplicity, **a set of probes classified as "species-specific" in the 2nd criteria that passed the threshold *x *are **hereafter named "SSP *x*"**. Different thresholds were tested, and the optimal threshold was selected according to the lowest variance of resulting RNA ratios.

### Stromal cell to cancer cell mRNA ratio and its validation (Figure 1, step C-1)

Evaluation of the stromal cell to cancer cell mRNA ratio, hereafter named as S/C ratio for simplicity, was independently carried in two steps. The samples of the *in vitro *biological experiments data for which the proportion of mouse to human RNA is designed and known (GSE23377) were used to validate the mouse to human RNA ratio metric. This metric then serves as a proxy for stroma to cancer cell RNA ratio in xenograft models consisting of human cancer cell tumors harvested from mice hosts.

### Gene ontology enrichment of cross-species hybridizing probes (Figure 1, step C-2) and visualization

After masking the top 5% of the most likely probes to exhibit CSH, KEGG/GO enrichment analysis was performed among the genes targeted by probes on the custom array using the conditional hypergeometric test in the Bioconductor *GOStats *package[26]. For comparison, the same pathway/GO enrichment assessment was performed on the commercial CSH probes, for the human and mouse species separately. The resulting p-values were adjusted for multiple testing by FDR[27] and pathways/GO terms with less than 500 gene members were counted [28]. Hexagon plots were conducted using the R package *hexbin *with default parameters.

## Results

### Human tumor and mouse xenograft cross-species hybridization of commercial arrays

Our previous study reported interspecies genetic differences and elucidated stromal microenvironment signals from probes on human arrays unintentionally cross-hybridizing with mouse homologous genes in xenograft tumor models (Figures 1, 3, 4 in the publication [17]). By identifying CSH probes from sequence alignment and CSH experiment for the human whole-genome arrays, deregulated stromal genes can be identified and then their biological significance confirmed by the laser capture microdissection of stromal cells from tumor specimens [17]. In this study, within Agilent 44k whole genome human arrays and mouse arrays, 4,259 commercial probes (2,104 human, 2,155 mouse) pertaining to 3,550 distinct genes cross-hybridized above our optimal threshold (highest 5% CSH) were annotated as cross-species cross-hybridizing probes.

### Design of the dual species H&M array

Using our computational cross-species hybridization algorithms and the initial biologic experiment (**Methods**, 5% threshold CSH), we identified 4,259 commercial probes as cross-hybridizing (2,104 human, 2,155 mouse) corresponding to 3,550 distinct genes. Therefore, we designed a custom 2x105k array (H&M array, Agilent custom platform) combining all probes from both the human and mouse Agilent 44k commercial whole genome arrays (human: 41,000 probes; mouse: 41,174 probes). In addition, the H&M array contains new species-specific probes we designed: 5.5k human and 5.2k mouse (Additional file 1**- Supp. Table S2**), covering 30k distinct human targets and 32k mouse targets (Refseq IDs), respectively.

### Analysis and annotation of the H&M arrays

A total of 87k probes were included on the H&M platform version 1, containing all commercial human and mouse probes, and 3,608 newly custom designed probes (Additional file 1**- Supp. Table S2)**. Annotation for version 1 of the array was conducted by comparing the results from the initial *in vitro *biological experiment with the CSH theoretical predictions of probes on the H&M array. The optimal biological GS of CSH as observed in the *in vitro *experiments was defined by comparing the optimal CSH theoretical prediction model with the most ideal parameters using a threshold of 18% for mouse probes [17] and 20% for human probes. Meanwhile, the optimal parameters for BLASTn result interpretation were selected as previously published (Figure D in Supplementary Methods of our previous publication [17]), where the sequence alignment derived from BLAST depended on the length of the alignment (varying from 1 to 54) and the BLAST mismatch number (varying from 1 to 12).

We further sought to incorporate theoretically predicted and experimentally verified CSH probes to the array. As illustrated in Figure 1 and Additional file 1**- Supp. Table S2**, H&M array version 2 included 7.1k new custom probes, containing 5k probes designed from experimentally annotated CSH probes in version 1 of the platform, in addition to 2.1k probes that the Agilent protocol suggested as acceptable but whose theoretical prediction resulted in CSH. Together with the 3.6k custom probes designed in version1 array, the total 10.7k custom probes covered 16% (5.3k) of human genes and 15% (5.1k) of mouse genes (Additional file 1**- Supp. Table S2**). The set of BLAST prediction parameters derived from the initial *in vitro *biological experiment (**Methods, step B3**) performed better in predicting CSH probes than a similar set of parameters with shorter mismatch lengths **(Methods, step B2)**. Two GSs for CSH (Table 2: GS2 and GS3) performed similarly in the initial *in vitro *experiment [17], making it necessary to validate the initial experiment. We found that biological GS3 that yielded a higher F-score than biological GS2. The former used relative CSH expression values (i.e. the ratio of the absolute CSH expression when exposed to the RNA of the other species compared with the expression when exposed to the RNA of the same species), while the latter used absolute CSH expression values due to the larger number of genes with multiple probes in version 2 of the H&M array. A total of 4,644 probes on the H&M array (version 2) were annotated as CSH, which comprised the top 5% relatively erroneously expressed (relative CSH) probes achieving the highest recall when compared with CSH theoretical prediction in Model 1-1 (Additional file 1**- Supp. Table S2**). This threshold of 5% was derived from H&M array version 1 probes, as version 2 included all version 1 probes. The estimated recall for mouse CSH probes was 25.6% (i.e. 25.6% probes were truly CSH among all annotated "CSH" mouse probes) and precision was 13% (13% of all theoretically predicted CSH mouse probes are annotated by our biological GS), while the annotated recall and precision was 27% and 11%, respectively, for human CSH probes. Additionally, our extensive annotation stratified all probes on H&M array version 2 into ten levels according to their probability of CSH, thus providing array users with more flexibility to disregard CSH probes in their analyses.

### Validation of H&M array version 1 annotation and reduction of CSH

**Figure 2:**
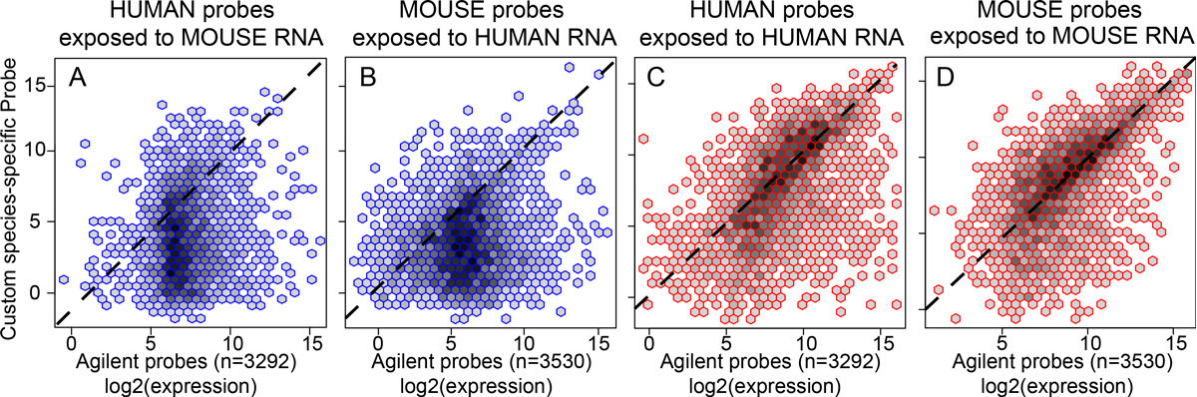
**Comparison of expression of custom designed species-specific probes (Y axis) against the Commercial Agilent 44k array probes (X axis)**. The log2 expression of custom species-specific probes (Y axis) was plotted against that of their Agilent counterpart targeting the same gene (mean expression value, n = 3 arrays). The dashed diagonal corresponds to equal expression between the two types of probes. The density distribution of the number of pairs of probes is shown as hexagons with increased color intensity. As shown in Panels A and B, the human custom species-specific probes obtain a *lower expression than their Agilent counterpart *with mouse RNA and the similar results for mouse probes (4.5 median fold reduction, 95% CI: 4.2, 4.9 for human probes; and 9.4 median fold reduction, 95% CI: 8.8, 10.3 for mouse probes; P < 2.2x10^-16^ for both; Non-parametric Mann-Whitney U tests). Panels C and D show that these species-specific probes of the H&M array perform as well on average as those Agilent probes when exposed to the intended RNAs. Human-mouse gene cross-hybridization patterns we discovered are provided in **Dataset S1 **(http://lussierlab.org/publications/HsMm_array).

The reduction in CSH was further shown by an analyses illustrated in Figure 2, which shows the comparison of version 1 custom designed 3,292 human probes and 3,530 mouse probes (y-axis) with the pair-wise probes on the Agilent commercial array (x-axis) for cross-species expression using the validating biological experiment. In contrast, when exposed to the RNA of the correct species, there was no significant difference between custom newly designed probes and the corresponding probes on the Agilent 44k whole genome array (Figure 2, **panels C-D**). Additionally, version 1 annotated CSH probes were significantly over-represented among the version 2 annotated CSH probes (p < 2x10^-16^, OR = 3.3). Here, the overlap between the annotated CSH probes demonstrates the robustness of our design to identify the CSH probes in a xenograft model.

### Estimation of stromal cell to cancer cell mRNA ratio

**Figure 3:**
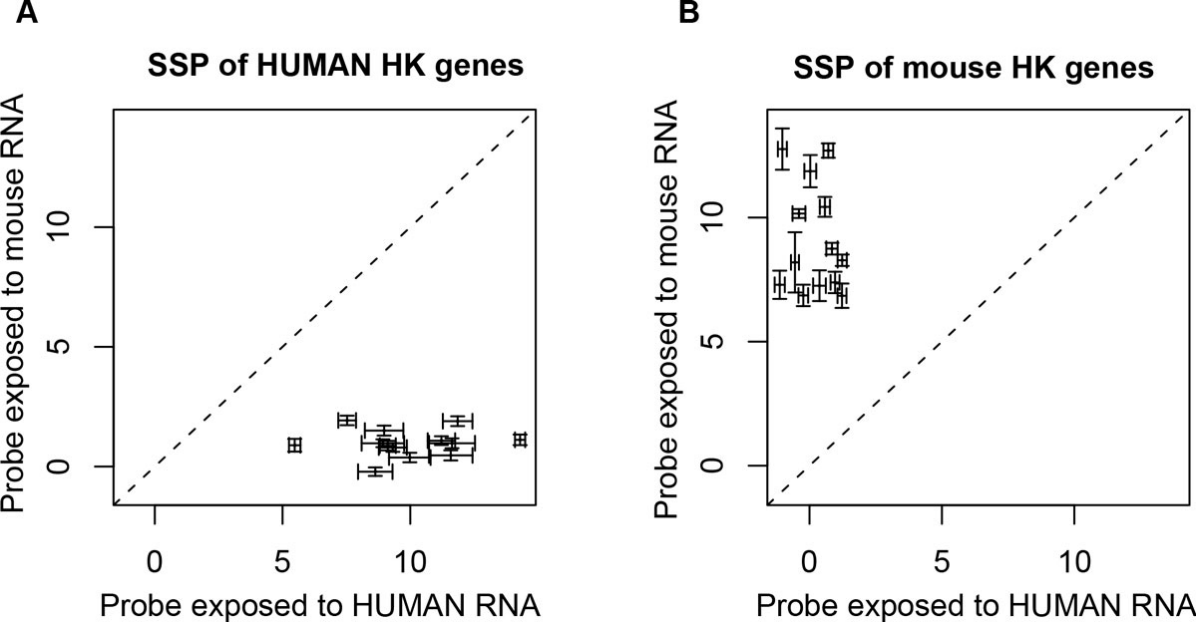
**Species-specific probes of pair-wise human and mouse homologous housekeeping genes**. The expression of 26 species specific probes (SSPs) corresponding to 13 pairs of human and mouse homologous housekeeping genes (HK) are shown according to two experimental conditions: exposure to the H&M array to Human RNA (X axis) or mouse RNA (Y axis). Each point is the mean expression value of 3 arrays and the whiskers are the 95% confidential intervals (n = 6 arrays total). A median fold change for the 13 pairs of SSPs resulted in a 10.4 fold change for human SSPs (panel A) and 9.7 fold change for mouse SSPs (panel B). Homologous genes utilised: *RNASEH1, PMPCB, SFRS8, PRDX6, TRAPPC4, MATR3, NIPA2, MRPL49, NOL7, VPS26A, HNRPDL, RPL39, OSBP*.

In an *in vitro *validation experiment for stromal to cancer cell ratio estimation, H&M arrays were separately exposed to human and mouse RNAs (Table 1). The ratios of the normalized expression between human SSPs and mouse SSPs targeting homologous housekeeping genes listed in Figure 3 were calculated.

As shown in Figure 3, the expression values of human probes exposed to only human RNA were larger than when exposed to only mouse RNA (Figure 3, **panel A**, median ratio between human and mouse probes is 10.4), and as shown in **Panel B**, the expression of mouse probes exposed to only mouse RNA was larger than that when exposed to only human RNA (median ratio between mouse and human probes is 9.7). Changing the thresholds to define an individual probe as species-specific (**Method**, Step C-1) to include from 10 to 50 pairs of human and mouse SSPs provided a similar result, however the expression ratio between human and mouse probes tended to be unchanged when all non-SSP were included (data not shown). These results suggested that the proportion of each species RNA can be estimated by the species-specific human and mouse probes of the pair-wise homologous housekeeping genes.

### Stromal cells to cancer cell mRNA ratio (S/C ratio) validation

**Figure 4:**
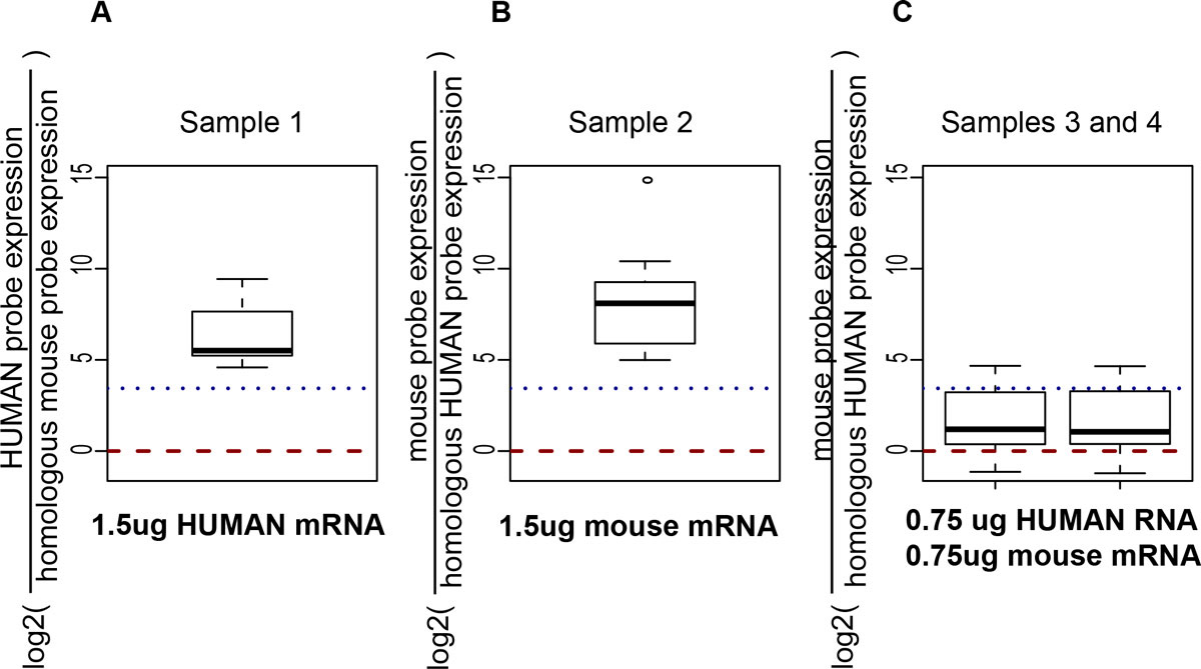
**Validation of cancer vs stromal RNA ratio using the expression of pair-wise human and mouse homologous housekeeping genes measured by species-specific probes**. The log2 expression ratio between human SSPs and mouse SSPs were plotted in three cases using the initial experimental data, when probes were exposed to: A) only human RNAs, B) only mouse RNA, and C) both human and mouse RNA. Consistent with the results shown in Figure 3, the expression ratios were larger than 10 (log2(10)=3.3, the dashed blue lines) when SSPs were exposed to the indented RNA only, while the 75th quantile of expression ratios were between 0 and 10 when exposed to equally human and mouse RNAs (panel C).

Validations for our S/C ratio estimation was performed with an *in vitro *biological experiment, where the stromal cell proportions were controlled using known quantity of RNAs as shown in Table 1. Using the *in vitro *CSH experimental data, where two arrays containing both human and mouse probes were exposed to equal quantities of human and mouse RNAs (**GSE23377**), the ratios of 13 pairs of homologous housekeeping gene expression were larger than 10-fold when exposed to the intended RNA of one species only (Figure 4**, panels A,B**), while was between 0 and 10 when exposed to an equal mix of human and mouse RNAs simultaneously (Figure 4**, panel C**, Table 1). The higher expression of mouse SSPs in panel B indicated a better mouse RNA quality than human RNA.

### GO and KEGG pathway enrichment analysis of cross-species hybridizing probes

To control for CSH of probes in GO and KEGG pathway enrichment analyses, we masked the 2,324 CSH human probes on the custom H&M array, and observed no KEGG pathways and only 1 GO molecular function (*RNA binding *including more than 600 gene members) significantly (FDR <5%) over-represented among the 44,144 human SSPs (targeting 30k Refseq IDs, 97% coverage of Agilent whole human genome array). We found no bias in GO or KEGG for the mouse probes of the custom H&M array. Notably, gene ontology and pathway biases exist among the CSH probes on commercial single-species arrays, and should be accounted for on-going and future xenograft tumor studies. Here we are the first to report comprehensive analysis of CSH probes between human cancer cells avoiding these biases. We identified eight significantly enriched (FDR<5%) KEGG pathways (seven human genes and one mouse gene targeted only by CSH commercial probes), eight GO molecular functions (all among human genes), and conclusively, stromal functions detected when using a dual species array for xenograft tissues due to gene expression in mouse stromal tissues, rather than in human cancer cells (data not shown). Further, CSH probes confound the results of microarray analyses of the metabolism pathway of xenobiotics by cytochrome P450 when xenograft tumor models were used due to the highly conserved nature of many cytochrome P450 genes (data not shown). Using Agilent 44k human arrays, Sugawara *et al*. reported 28 genes demonstrating higher induction in xenograft tumors compared to the cells in culture medium[15]. Our independent analysis revealed that 10 of these 28 previously reported genes are targeted by commercial CSH probes (data not shown). Additionally, we observed that the cytochrome P450 pathway involved in xenobiotic metabolism is enriched (cumulative hypergeometric pvalue = 1x10^-5^) in the CSH probes found in the list of 28 genes (data not shown), as would be expected for our xenograft model. Other significant pathways and functions listed that suggest relevant stromal functions include inflammatory signalling through the complement system, focal adhesion and tight junctions involving transmembrane proteins mediating intra-membrane and paracellular diffusion, as well as motor activity and actin binding, and thus possibly mediating invasion and metastasis processes. These results indicated that the design of dual-species arrays and the identification of CSH probes are critically important for studies involving highly conserved genes and pathways in xenograft models.

## Discussion

We established that overlooking CSH probes in xenograft models using commercial human arrays confound incorrect attribution of deregulated stromal genes to cancer cells. To increase the capability of analysing molecular changes in the stromal compartment as well as the interactions between cancer cells and reactive tumor stroma in xenograft modelling, we designed the first custom genome-wide human-mouse microarray, and biologically validated it by observing an overall reduction of the CSH (4.5 median fold reduction for human probes; and 9.4 median fold reduction, for mouse probes; p < 2x10^-16^, Mann-Whitney test). By design, we select a framework of well-established computational and biological methods to identify cross-hybridizing probes, construct a cross-hybridizing map, and identify new SSPs. Here, we provide a combined description of the interplay between the computational predictions of CSH of probes using BLASTn and observation of biological CSH that were utilized to develop an approach stratifying probes in terms of their propensities for cross-hybridization. We also implemented a rigorous rationale for the selection of a threshold needed to redesign the cross-hybridizing probes and offer the foundational datasets used for conducting analyses over a dual-species array: a comprehensive map of cross-hybridizations annotated with both sequence alignment and biological data intended to increase the accuracy and efficiency of measurements in tumor xenograft models. Additionally, we developed a novel method to estimate the mRNA ratio between mouse and human mRNA as a cellularity ratio, thus providing an alternative to conventional histological counts of stroma vs. cancer cells in xenograft models. Of note, the H&M array could also be utilized in single species experiments, as long as the probes of that species are selected. This information is clearly available in the "species" column of **Dataset S1**. Further, for comparison of H&M array results against previously published dataset using single species commercial Agilent arrays, the H&M probes corresponding to the commercial platforms are annotated in the column "designer" of **Dataset S1**.

One limitation of the current study is that grouping all of the stromal cell types into one category may be too strong of a simplification of the complex cellularity of the host tissue. For instance, changes in the presence of certain types of stromal cells have profound effects on the status of the tumor, and stromal cells are thought to genomically "co-evolve" along with cancer cells during tumor progression [29]. Also, the xenograft model involves immune deficient mice, and therefore cannot be used to understand much about the stromal immune cells and associated pathways that participate in regulating the tumor in the xenograft models. However, xenograft models re-constituting bone marrow cells from syngeneic donors may alleviate this limitation in future studies. Further, with the advent of RNA-sequencing, the utility of the present study may reside in our supplemental datasets that allow to reanalyze the species-specific probes of commercial arrays for comparison with RNA-seq experiments or to inform assembly algorithms of RNA-seq in xenograft models. Another limitation is that we have not considered non-coding RNAs in this study.

## Conclusion

There is ample indication that the issue of CSH is overlooked in xenograft modelling, as the majority of tumor xenograft studies utilize gene expression over human arrays indiscriminately with no correction for homologous probes [30-35], leading to the incorrect attribution of deregulated stromal genes to cancer cells. Furthermore, commercial human and mouse arrays are shown to contain a large number CSH probes, suggesting that commercial designs did not consider the potential reuse of these arrays for xenograft modelling. While the computational methods and biological validations are straightforward and rely on well-established methods, the originality of this study resides in (i) appropriately computationally modelling the deceptively simple source of CSH (homologous genes) - a problem that has nonetheless been overlooked by microarray corporations and by many xenograft biologists in their array-related publications, in (ii) providing well-validated and novel work products: a detailed cross-hybridization map of human and mouse probes, as well as a cost-effective and superior array for expression analyses of human xenograft models, and in (iii) offering a new *in silico *metric for estimating the cellularity ratio of cancer over stroma that should in principle be applicable to RNAseq transcriptomes as well. This study's findings will improve our capability of investigating and interpreting the interactions between human cancer and mouse stromal cells in xenograft modelling. Together, these technological improvements will allow effective interrogation of cancer cell-reactive stroma interactions during cancer initiation, progression and response to therapeutic interventions.

## Abbreviations

LCM, laser capture microdissection; CSH, Cross-Species Hybridization; GO, Gene Ontology; H&M array, Human-Mouse dual-species array; KEGG, Kyoto Encyclopedia of Genes and Genomes; GS, Gold Standard; SSP, Species Specific Probes; HK, housekeeping genes; FDR: False Discovery Rate; CI, Confidence Interval; BLAST, Basic Local Alignment Search Tool.

## Competing interests

The authors declare that they have no competing interests.

## Authors' contributions

YAL supervised the entire work; XY designed and conducted the analyses and annotation of data; YH conducted the online custom Agilent array design; YL performed the BLASTn programming; YAL, YH and ER contributed to the biological interpretation of the results; YAL, XY, HY, ER, VG and AI wrote and revised the manuscript.

## Supplementary Material

Additional file 1 **Supplement Tables and Figures**. This document contains the two supplement tables (Tables S1, S2) and two supplement figures (Figures S1, S2). • Table S1 **- **Data resources used in this study. • Table S2 **- **Summary of probes on H&M array version 2 • Figure S1 **- **Optimization of Models identifying cross-species hybridizing probes. • Figure S2 **- **BLAST parameter selection based on higher F-scores. Click here for file

## Declarations

Publication for this article has been funded by grants 1U54CA121852, UL1 RR024999-03, K22 LM008308, R21 CA167305-01A1. This work was supported in part by the UA Cancer Center grant (NCI P30CA023074), the UA BIO5 Institute, and the UA Clinical & Translational Science Institute of the University of Arizona.

This article has been published as part of *BMC Medical Genomics *Volume 7 Supplement 1, 2014: Selected articles from the 3rd Translational Bioinformatics Conference (TBC/ISCB-Asia 2013). The full contents of the supplement are available online at http://www.biomedcentral.com/bmcmedgenomics/supplements/7/S1.
