# Supplementary material for: In Silico cancer cell versus stroma cellularity index computed from species-specific human and mouse transcriptome of xenograft models: towards accurate stroma targeting therapy assessment
Source: BMC Med Genomics. 2014 May 8;7(Suppl 1):S2. doi: 10.1186/1755-8794-7-S1-S2 (PMC4101338; doi:10.1186/1755-8794-7-S1-S2)

In Silico Cancer Cell versus Stroma cellularity index computed from species-specific human and mouse transcriptome of xenograft models: towards accurate stroma targeting therapy assessment

Table S1 - Data resources used in this study.

	Content	Data source	Data version
1	Probe annotation for Agilent 44k whole human genome array	GPL6480 from GEO	on Sep., 2009
2	Probe annotation for Agilent 44k whole mouse genome array	GPL7202 from GEO	on Sep., 2009
3	Possible mouse targets of probes in human array	Agilent company (**Dataset S2**)	May, 2008
4	Possible human targets of probes in mouse array	Agilent company (**Dataset S3**)	May, 2008
5	The mouse homolog of human genes	NCBI (ftp://ftp.ncbi.nih.gov/pub/HomoloGene)	Build63
6	The mapping between Entrez gene ID and RefSeq ID annotated for each probe for enrichment analysis	Bioconductor database org.Hs.eg.db and org.Mm.eg.db	Version 2.3.6
7	The GO annotations for both human and mouse genes for enrichment analysis	Bioconductor database GO.db	Version 2.3.5
8	The KEGG annotations for both human and mouse genes for enrichment analysis	Bioconductor database KEGG.db	Version 2.3.5
9	Evidence based selection of human and mouse housekeeping genes	http://www.plosone.org/article/fetchSingleRepresentation.action?uri=info:doi/10.1371/journal.pone.0000898.s001 [22,23]	Oct. 2010

Table S2 - Summary of probes on H&M array version 2

		H&M Array 2		CSH		After Masking CSH	
		probes	genes*	probes	genes*	probes	genes*
**H&M**	total	92852	63147	4644	4165	88208	61103
	commercial	82155	63143	4259	3944	77896	60347
	custom	10697	10360	385	379	10312	9991
**Hs**	total	46468	30360	2324	2009	44144	29353
	commercial	40990	30359	2104	1891	38886	28933
	custom	5478	5273	220	217	5258	5061
**Mm**	total	46384	32789	2320	2157	44064	31751
	commercial	41165	32785	2155	2054	39010	31415
	custom	5219	5089	165	162	5054	4931

* The number of genes was counted by the unique Refseq IDs.
Legend: CSH = Cross-Species hybridization

In Silico Cancer Cell versus Stroma cellularity index computed from species-specific human and mouse transcriptome of xenograft models: towards accurate stroma targeting therapy assessment

Figure S1 - Optimization of Models identifying cross-species hybridizing probes.

Calculation of mismatches of the blast alignment was determined using the optimal F-Score while varying systematically two variables: the % of tolerated CSH according to the gold standard and the model (see **Table 2).** Using the validation CSH expression experiment data, the comparison for human probes is shown on the left and for mouse probes on the right. Specifically, erroneous expression values refer to the expression values of mouse probes exposed to human RNAs, or the expression values of human probes exposed to mouse RNAs. The figure shows that the CSH theoretical prediction Model 1-1 (red) is superior to Model 1-2 (black dotted line). Of note, parameters are given by **Table 2** in the manuscript.

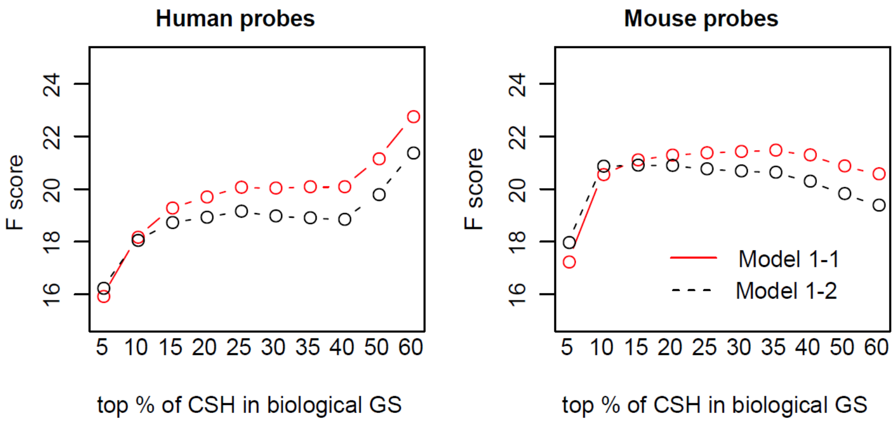

Figure S2 - BLAST parameter selection based on higher F-scores.

In the graphs below, the comparison for the nucleotide sequence alignment > 50 nucleotides using mouse probes is shown as a demonstration. Compared with the optimal biological GS, for all systematically calculated F-scores of different tolerant mismatch numbers from 1 to 12, the highest F-score appeared at mismatch <11 for all alignments with >50 comparisons. Similar processes were conducted for the other regions of BLAST alignments listed in Table 2, and Model 1-1 resulted in the best combination of parameters derived from the initial experiment (Max F-score).

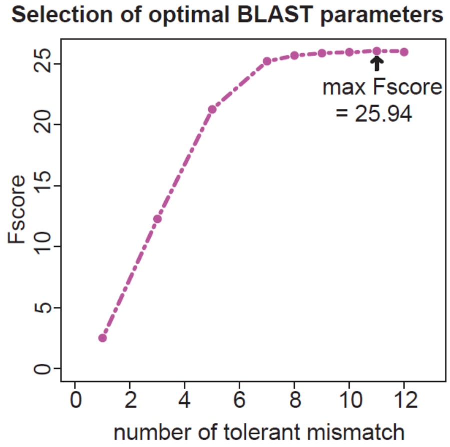

Supplement: Additional file 1 — Supplement Tables and Figures. This document contains the two supplement tables (Tables S1, S2) and two supplement figures (Figures S1, S2). • Table S1 - Data resources used in this study. • Table S2 - Summary of probes on H&M array version 2 • Figure S1 - Optimization of Models identifying cross-species hybridizing probes. • Figure S2 - BLAST parameter selection based on higher F-scores. [file 1755-8794-7-S1-S2-S1.PDF]
